# Virtual reality in functional neurological disorder: a theoretical framework and research agenda for use in the real world

**DOI:** 10.1136/bmjno-2023-000622

**Published:** 2024-07-05

**Authors:** David Brouwer, Hamilton Morrin, Timothy R Nicholson, Devin B Terhune, Michelle Schrijnemaekers, Mark J Edwards, Jeannette Gelauff, Paul Shotbolt

**Affiliations:** 1 Department of Neurology, Amsterdam UMC Location AMC, Amsterdam, The Netherlands; 2 Neuropsychiatry Research and Education Group, King's College London Institute of Psychiatry, Psychology & Neuroscience, London, UK; 3 South London and Maudsley NHS Foundation Trust, London, UK; 4 Department of Psychology, King's College London Institute of Psychiatry, Psychology & Neuroscience, London, UK; 5 Computational Science Lab, University of Amsterdam, Amsterdam, The Netherlands; 6 Department of Psychological Medicine, King's College London Institute of Psychiatry, Psychology & Neuroscience, London, UK

**Keywords:** FUNCTIONAL NEUROLOGICAL DISORDER, MOVEMENT DISORDERS, NEUROPSYCHIATRY, ATTENTION, REHABILITATION

## Abstract

Functional neurological disorder (FND) is a common and disabling condition at the intersection of neurology and psychiatry. Despite remarkable progress over recent decades, the mechanisms of FND are still poorly understood and there are limited diagnostic tools and effective treatments. One potentially promising treatment modality for FND is virtual reality (VR), which has been increasingly applied to a broad range of conditions, including neuropsychiatric disorders. FND has unique features, many of which suggest the particular relevance for, and potential efficacy of, VR in both better understanding and managing the disorder. In this review, we describe how VR might be leveraged in the treatment and diagnosis of FND (with a primary focus on motor FND and persistent perceptual-postural dizziness given their prominence in the literature), as well as the elucidation of neurocognitive mechanisms and symptom phenomenology. First, we review what has been published to date on the applications of VR in FND and related neuropsychiatric disorders. We then discuss the hypothesised mechanism(s) underlying FND, focusing on the features that are most relevant to VR applications. Finally, we discuss the potential of VR in (1) advancing mechanistic understanding, focusing specifically on sense of agency, attention and suggestibility, (2) overcoming diagnostic challenges and (3) developing novel treatment modalities. This review aims to develop a theoretical foundation and research agenda for the use of VR in FND that might be applicable or adaptable to other related disorders.

## Introduction

Functional neurological disorder (FND) is frequently encountered by neurologists.^1^ FND encompasses a wide range of neurological symptoms and includes several subtypes, including functional movement disorder (FMD), which in turn is one of the most common movement disorders,^2^ and persistent perceptual-postural dizziness (PPPD).^3^ Motor symptoms in FND may include tremor, dystonia, myoclonus and/or paresis. Clinicians can distinguish these symptoms from other neurological disorders by using positive rule-in criteria, such as entrainment in tremor and Hoover’s sign in paresis, alongside more general characteristics such as distractibility and suggestibility.^4 5^

As FND falls into the gap between physical and mental health, historically, the disorder has remained relatively poorly understood and under-researched. However, over the last 10–15 years, there has been considerable progress in FND research across mechanisms, diagnosis and treatment.^6^ In particular, there has been a shift from purely psychological theories regarding conversion of stress to a broader biopsychosocial framework emphasising the importance of biological risk factors and mechanisms, and their compatibility and interaction with cognitive as well as psychological mechanisms as part of more nuanced multifactorial models.^7 8^ Despite these promising developments, there is still significant uncertainty in the field of FND, most evidently demonstrated by limited evidence-based treatments.^7^

There is increasing interest in and use of virtual reality (VR) technology across a wide range of neurological and psychiatric disorders.^9^ Despite there being several theoretical reasons why VR could be of utility in FND, there has been little research in this area. In this review, we provide a theoretical framework supporting the implementation of VR-based approaches in FND, with a primary focus on FMD (ie, motor FND) to illustrate and underpin the potential of VR which could be applied and adapted to most if not all FND subtypes.

## Current applications of VR

**Figure 1:**
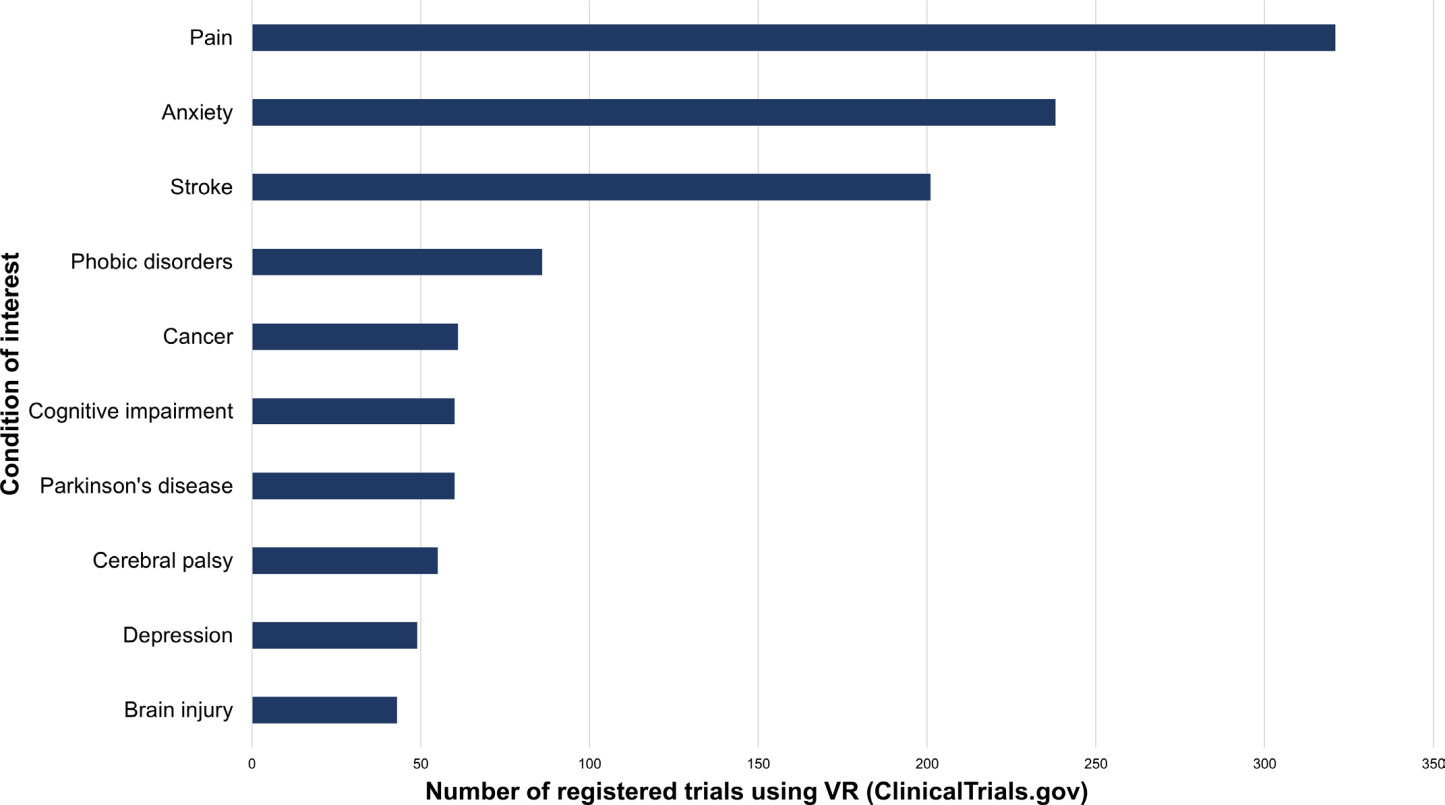
Number of registered trials on ClinicalTrials.gov^12^ using VR shown for the 10 most commonly mentioned conditions of interest. VR, virtual reality.

Over recent decades, VR has emerged as a safe and engaging tool leading to significant advances across medicine from direct therapeutic applications delivered in VR to using VR for training and educating healthcare professionals and investigating mechanisms of disorders.^10^ VR-based approaches hold particular appeal due to their ability to better maintain attention to tasks, replicate real-world rehabilitative interventions in a clinical setting and create novel scenarios not possible in the real world.^11^ In addition, studies of neurological and psychiatric conditions comprise a considerable proportion (70%) of clinical trials using VR, of which there are to date over 2000 registered on ClinicalTrials.gov^12^ (figure 1).

### Neurology

In neurology, most VR interventions are rehabilitative^9^ and largely target motor symptoms, replicating conventional rehabilitation programmes in a VR environment.^13^ In stroke, VR interventions have demonstrated effects on functional motor outcomes comparable with those of conventional rehabilitation, and when applied as adjunct have significantly augmented effects on upper limb function and manual dexterity.^13 14^ Furthermore, VR rehabilitation displayed comparable outcome effects on gait and balance in Parkinson’s disease.^15^

In addition to motor symptoms, VR rehabilitation has been applied in cognitive disorders, demonstrating moderate-to-large effects on global cognition, attention and memory in patients with mild cognitive impairment (MCI) or dementia.^16^ Besides therapeutic purposes, VR has been used in the diagnosis of MCI, showing substantial detection performance.^17^

### Psychiatry

In psychiatry, VR has typically been used as a method of simulation and/or distraction.^18 19^ VR exposure therapy is the most studied VR intervention and uses simulation^20^ to allow controlled exposure to a wide range of situations, for example, for specific phobias, with preliminary evidence suggesting comparable efficacy with in vivo exposure.^21^ Just recently, the first VR treatment was added to the National Institute for Health and Care Excellence guidelines, as treatment for agoraphobia.^22^ Additionally, there are numerous VR training programmes that involve social interaction, such as social skills training in patients with psychosis or autism spectrum disorder.^20 23^ There are also several VR programmes that facilitate stress reduction through distraction.^19^ In addition, more experimental VR interventions are being developed^24^; for example, virtual body ownership illusions altering distorted body perceptions as a new treatment modality for eating disorders and obesity.^25 26^

For diagnosis, VR shows promise due to its capacity to simultaneously provoke and measure psychiatric symptoms.^27^ This is for instance done by measuring hyperactivity in attention-deficit/hyperactivity disorder (ADHD) by capturing bodily movements, and by assessing paranoia and social functioning in psychotic disorders.^18 27^

In both neurology and psychiatry, most VR interventions are treatment focused and based on conventional interventions. However, VR also allows for novel interventions that would be difficult or impossible in real life.

### Pain

There is a significant potential role for VR in pain management, which is of particular relevance given the high prevalence of comorbid pain in FND.^28^ Distraction is the main mechanism, though other targets, such as improving mood or teaching psychological coping skills, exist.^29^

In acute pain, VR consistently demonstrated moderate effect sizes in pain reduction (varying depending on population, condition and timing).^30^ In chronic (musculoskeletal and/or neuropathic) pain, there is preliminary evidence suggesting significant effects on pain severity, with more research needed to support long-lasting effects.^30 31^

Several studies have explored the use of VR in fibromyalgia and have found positive impacts on quality of life and pain.^32 33^ One study that employed a progressive body invisibility illusion found strength of embodiment was positively associated with body perception disturbances and negatively with symptom intensity.^34^ In addition, VR mirror visual feedback therapy, which is used to create illusions normalising the distorted representation of the affected body part, has demonstrated potential effects in phantom pain^35^ and complex regional pain syndrome,^36^ in addition to its rehabilitative purposes in patients who had a stroke.^37^ However, these studies have not included placebo control conditions and multiple lines of evidence challenge the presumed mechanistic basis of these effects.^38 39^

### Functional neurological disorder

**Figure 2:**
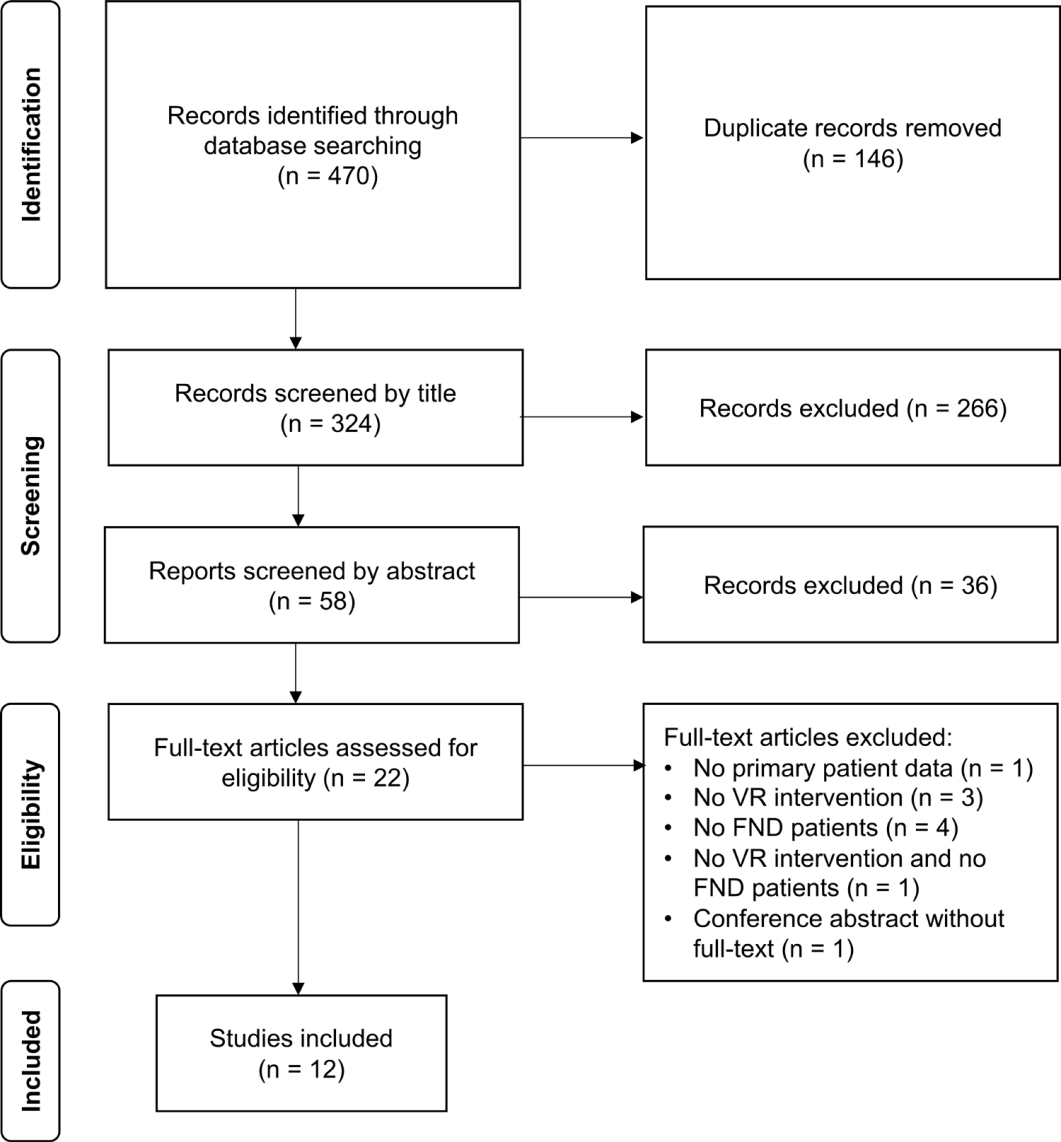
Preferred Reporting Items for Systematic Reviews and Meta-Analyses flow diagram for systematic search of literature on VR in FND. FND, functional neurological disorder; VR, virtual reality.

We systematically searched for both published studies and clinical trials currently being conducted on VR and FND (see online supplemental methods for search strategy). Our search identified 12 published studies on the application of VR in FND (figure 2), of which four focus on FMD specifically^40–43^ (table 1), seven focus on PPPD^44–50^ (table 2) and one on dissociative amnesia^51^ (table 1). Two trials currently in progress were also identified (table 1). In terms of study types, there were: four mechanistic functional MRI (fMRI) studies, three randomised controlled trials, three case–control pilot studies, one retrospective study and one case report.

10.1136/bmjno-2023-000622.supp1 Supplementary data

**Table 1:** Summary of published and unpublished research on applications of VR in motor and cognitive FND

Reference	Study type	Methodology	Findings	Relevance
Functional motor/movement disorder (FMD)				
Nahab *et al* 40	Mechanistic fMRI study	Behavioural experiment using virtual hand illusion in individuals with FND (n=21) vs healthy controls (n=20), testing sense of agency over virtual hand that would or wouldn’t follow their own movements	Altered activity in regions associated with sense of agency on fMRI—right dorsolateral prefrontal cortex and pre-supplementary motor area did not respond differentially to loss of movement control.	Provides a paradigm for altering sense of agency in patients with FND using VR by altering synchronicity between real and virtual hand movements.
Bullock et al*^41^	Single-blind randomised controlled pilot study (midpoint data)	Participants in the treatment arm (n=7) received 8 sessions of VR mirror visual feedback therapy. Starting from session 4, participants additionally received VR exposure therapy based on self-identified FND triggers. The primary outcome measure was symptom severity.	86% completion rate (n=12/14). No side effects or adverse events. Symptom frequency and level of disability (Oxford Handicap Scale^115^) were reported but not statistically analysed.	Midpoint results support general feasibility in the FND patient population.
Nguyen *et al* 42	Case report of VR physiotherapy	Use of three commercially available VR games to encourage either upper limb, lower limb or fine motor movements. Use of objective movement tracking application.	A 5-fold increase in total movement from physiotherapy rehabilitation VR sessions 1–2. With reduction in wheelchair requirement, improved balance and smoother gait.	Highlights utility of objective movement tracking in 3D space.
Gandolfi *et al* 43	Case–control pilot study	Comparison of postural parameters in FMD (n=17) and healthy controls (n=19) under four attention-demanding conditions: simple fixation task (1) in a real room and (2) in 3D VR room-like condition; complex fixation task in a 3D VR city-like condition (3) avoiding distractors and (4) counting them.	Patients displayed reduced sway area and mediolateral centre of pressure displacement velocity dual task effect but only under most attention-demanding condition (4).	Preliminary evidence for potential utility of combined immersive VR environment with graded attention-demanding conditions in adapting postural control in FMD.
https://clinicaltrials.gov/study/NCT05086380	Treatment trial (no further details)	Transcranial magnetic stimulation or mindfulness-based stress reduction therapy to modulate sense of agency and well-being in FND (symptom type unspecified) and other neurological disorders.	Currently recruiting	The study references use of VR to examine movement patterns and symptoms, but no further methodological details are available in the published protocol.
Dissociative amnesia				
Weniger *et al* 51	Mechanistic fMRI study	10 women with dissociative amnesia, and 4 with dissociative identity disorder, and 14 healthy controls underwent fMRI while navigating a virtual maze, controlling movements with a joystick.	Individuals with dissociative disorders where not impaired in learning the virtual maze compared with controls and showed similar though weaker patterns of activity change during egocentric spatial learning.	Though the study population is not entirely homogeneous, this serves as an example of the use of VR to study attentional and visuospatial memory function in functional cognitive disorder.

*This study corresponds to the second trial found in the ClinicalTrials.gov database (https://clinicaltrials.gov/study/NCT02764476).

3D, three-dimensional; fMRI, functional MRI; FND, functional neurological disorder; VR, virtual reality.

**Table 2:** Summary of published research on applications of VR in PPPD

Reference	Study type	Methodology	Findings	Relevance
Riccelli *et al* 44	Mechanistic fMRI study	VR rollercoaster with fMRI in patients with PPPD (n=15) and healthy controls (n=15). Comparisons between groups made for alteration in vestibular and visual cortical activity with vertical vs horizontal displacement.	Controls (but not patients with PPPD) displayed increased activity in anterior bank of central insular sulcus during vertical relative to horizontal motion. For the same comparison, dizziness handicap^116^ correlated positively with visual cortex activity in those with PPPD.	Provides some insight into PPPD functional alterations in brain processes that may affect balance control and reweighting of space–motion inputs to favour visual cues.
Passamonti *et al* 45	Mechanistic fMRI study	Secondary analyses of Riccelli *et al*, exploring relationship between neuroticism and introversion (Revised NEO Personality Inventory^117^)	Neuroticism positively correlated with inferior frontal gyrus activity, and enhanced connectivity between inferior frontal gyrus and occipital regions in patients with PPPD relative to controls during vertical vs horizontal movement.	Raises question on role of personality traits in activity and connectivity of neural networks mediating attention to visual motion cues during vertical motion in PPPD.
Lubetzky *et al* 46	Case–control pilot study	Changes in centre of pressure (COP) and head kinematics of people with PPPD (n=22) and healthy controls (n=20) were compared in response to different combinations of visual (static or moving stars) and cognitive perturbations (serial-3 subtraction) during a balance task.	Controls significantly increased all COP and head parameters with the cognitive task, whereas PPPD only increased COP mediolateral path and acceleration.	Some evidence for reduced movement with challenge in PPPD, particularly with regard to head position.
Aharoni *et al* 47	Case–control pilot study	Individuals with PPPD (n=22) and controls (n=29) performed a square-shaped fast walking task in VR under three conditions (empty train platform, people moving, people and trains moving).	State anxiety and simulator sickness did not increase following testing. There were no significant between-group differences in head kinematics. In high visual load conditions, high trait anxiety and longer timed-up-and-go (TUG)^118^ duration were associated with reduced anteroposterior and mediolateral range of movement in those with PPPD.	Some associations between head kinematics and self-reported and functional outcomes in those with PPPD were noted.
Choi *et al* 48	Randomised controlled trial	30 individuals with PPPD experienced a VR-based vestibular exercise, of whom 15 also received optokinetic stimulation (consisting of stars rotating around the yaw, pitch and roll axes for 3 min in each axis). This was conducted once a week for 4 weeks.	From baseline to 4 weeks, significant improvements in dizziness handicap inventory,^116^ activities of daily living (ADLs), Visual Vertigo Analogue Scale^119^ and TUG^118^ were noted. Only ADL and TUG showed significant improvement with optokinetic stimulation.	Preliminary evidence suggesting potential benefit of VR-based vestibular exercises in PPPD.
Mempouo *et al* 49	Retrospective study	Retrospective review of outcomes in 1000 patients with Situational Characteristic Questionnaire^120^ >0.9/4 indicating diagnosis of visual vertigo or PPPD who received VR-based therapy along with usual vestibular rehabilitation. Varied VR environments allowed identification of individual triggers and tailored desensitisation therapies.	Significant improvement was noted in Situational Characteristic Questionnaire (symptom severity),^121^ Nijmegen Questionnaire (hyperventilation),^122^ and Dizziness Handicap Inventory.^116^	Provides an example of a clinical physiotherapy service already making use of VR-based rehabilitation to provide more individualised treatment.
Yamaguchi *et al* 50	Randomised controlled trial	The VR group (n=12) included patients who underwent dual task, trunk balance training for PPPD for 100 tasks (10 min). This involved catching falling virtual objects and touching stationary virtual items in a video game-like training environment. The control group (n=14) received standard care.	The VR group displayed significant improvement in static and dynamic postural stability when comparing pre-single and post-single session outcomes. They also had significant improvement in Hospital Anxiety and Depression Scale^123^ and Niigata PPPD Questionnaire^124^ 1 week post-session.	Evidence for potential impact of dual task-based VR balance training on postural control and mood-related outcomes.

fMRI, functional MRI; PPPD, persistent perceptual-postural dizziness; VR, virtual reality.

VR applications varied though largely focused on attention. For FMD, VR mirror visual feedback training and exposure therapy were explored in a randomised controlled trial with midpoint data showing general feasibility (though efficacy remains to be determined).^41^ One case report made use of commercially available VR games involving physical activity for rehabilitation.^42^ Graded attentional dual tasks were also used in FMD to explore impact on postural stability.^43^ Similarly, PPPD studies (which comprise the bulk of the literature) largely used VR for balance training and vestibular rehabilitation via visual directional motion and rotation cues (eg, virtual rollercoasters, moving stars, trains) with several also making use of graded levels of dual-task attentional demands.^46 47 50^ The single study of functional cognitive disorder used a virtual maze to explore attentional and visuospatial learning in dissociative amnesia.^51^ Of note, none of the papers identified made use of augmented reality-based approaches. Overall, the current evidence is limited and warrants further exploration in larger studies.

## Mechanisms of FND

Many aspects of the mechanism(s) proposed to underlie FND indicate it is a disorder for which VR applications are potentially relevant. Current explanatory models recognise FND as a multifaceted disorder at the intersection of neurology and psychiatry, with intricate interplay between body and mind.^52^ One such explanation of FND symptoms is based on a Bayesian model of the brain,^53 54^ in which perception is described as a hierarchical process of inference, combining (top-down) prior expectations and (bottom-up) sensory input to minimise prediction errors. Functional symptoms then arise due to prediction errors that are not adequately updated, resulting in a multilevel cascade of dysfunction.^3^ Generally, it is understood that abnormal priors (prior expectations), which are given pathologically high precision (weight) through attentional processes, lie at the basis of these prediction errors.^53^ Overall, FND is then conceptualised as an abnormality in predictive processing.^3^

Some studies have generated empirical evidence supporting this framework; for example, Lin *et al* 55 provided evidence for the role of predictive processing abnormalities in patients with functional gait disorder, using the broken escalator phenomenon.^56^ Additionally, a recent study by Weissbach *et al* 57 further substantiated the role of altered perception–action integration processes in patients with FMD.

Our central premise is that VR, by creating a wide range of ways to study and modulate predictive coding abnormalities, could be used to directly manipulate and thus elucidate the hypothesised pathophysiological mechanisms underlying FND, thereby interrogating the Bayesian model of FND. For example, it can be used to conduct similar experiments to the broken escalator phenomenon, such as the VR plank falling experience, in which a decreased H-reflex is demonstrated during falling, implicating a direct link between the VR experience and motor response.^58^

The three most fundamental concepts in this Bayesian model, as components of predictive processes, are (1) attention, (2) sense of agency and (3) suggestibility.^53 59^ We explore each in turn as well as the potential interplay with VR.

### Sense of agency

#### Sense of agency in FND

A distorted sense of agency is understood to be a fundamental element of FND.^40 53^ Sense of agency is a cognitive phenomenon accompanying voluntary movements, and is defined as the feeling of being in control of one’s (motor) actions.^60 61^ It is related to sense of ownership, which refers to the feeling that your body parts, feelings or thoughts are your own.^62^ Theoretically, sense of agency is generated through the comparison of efferent predictions and afferent sensory feedback, that is, through sensorimotor integration.^63^ It consequently increases when there is a predictive match and diminishes with prediction errors.^3 60^

Although sense of agency is considered to be a crucial aspect of FND in general, its role is most evident in FMD, where patients experience physiologically willed movements as involuntary and show deficits in explicit motor control leading to difficulty performing simple motor tasks.^6 64^ Several experiments have been conducted on different implicit markers of sense of agency to substantiate its role in FMD. One such marker is temporal (action–outcome) binding, the perceived contraction of temporal interval between a motor action and its sensory effect, which is reported to be greater in healthy controls, but low in patients with FMD.^65^ Similarly, sensory attenuation, the phenomenon of reduced intensity of sensory perceptions when a movement is perceived as self-generated, was found to be reduced in patients with FMD compared with healthy controls.^66^ Despite this, a study of explicit sense of agency in individuals with FMD found that self-reported agency over tapping movements (Rubber Hand Illusion Questionnaire^67^) did not differ from that in healthy controls,^68^ suggesting a potential discrepancy between explicit and implicit sense of agency in this population.

#### Sense of agency in VR

Turning to VR, there is evidence regarding its potential to alter sense of agency.^61 69^ On a theoretical level, one can imagine how manipulation of sensory feedback may alter sense of agency by increasing or decreasing sensorimotor congruence.

Immersion in VR often leads to individuals experiencing a sense of ownership and agency regarding their virtual body and movements.^70^ This phenomenon was originally exemplified by the VR variant of the classic rubber hand illusion (RHI), where participants perceive ownership of a rubber hand being stroked synchronously with their real hand.^71 72^ Building on this, virtual limb and even virtual body illusions have been developed, where participants experience a complete virtual body as their own.^73 74^

One illuminating study using VR to alter sense of agency was conducted by Aoyagi *et al*.^61^ Subjects were instructed to make repetitive hand movements, following a designated circular trajectory. Delaying visual feedback resulted in a weakened self-reported sense of agency, whereas positional correction of the virtual hand towards the designated trajectory enhanced sense of agency.^61^ These results support the hypothesis that sense of agency can be altered by creating spatiotemporal discrepancies between self-generated movement and visual feedback.

#### Sense of agency in VR and FND

The foregoing results suggest that VR represents a viable method for altering sense of agency in patients with FND, thereby allowing investigation of the precise aetiological role of sense of agency in functional symptoms. Mechanistic research of sense of agency in FND using VR has been limited beyond the landmark study by Nahab *et al*,^40^ in which one notable finding was the tendency of patients with FMD to overestimate control over the virtual hand in comparison with healthy subjects. Indeed, when control over the virtual hand was entirely lost, patients failed to recognise that loss of control. In addition, by conducting the experiment during fMRI, the authors observed that the right dorsolateral prefrontal cortex and pre-supplementary motor area (regions involved in the cerebral network associated with sense of agency) were less responsive during loss of movement control in FMD compared with controls.^40^ This study lends weight to the involvement of an impaired sense of agency in FMD, but emphasises the complexity and uncertainty surrounding its precise role.

Compared with other means of studying sense of agency, VR is better equipped to manipulate sensory feedback, up to the point of manipulating perception of movement itself (which is particularly relevant in PPPD), suggesting multiple fruitful paths for future mechanistic research. First, it would be valuable to conduct experiments similar to those by Nahab *et al*,^40^ including varying the amount of perceived control by manipulating sensory feedback. The methods of sensory feedback manipulation could also be developed further, for example, by creating different spatiotemporal alterations, thereby offering a broad scope for conducting diverse experiments. Specifically, it would be useful to include functional symptom severity as outcome measures, to explore how variations in sense of agency influence symptoms. Second, comparable experiments in a third-person perspective may show promise, due to the potential for full-body ownership illusions to influence sense of agency.^24^ As first-person VR interventions seem limited to targeting upper limbs, third-person paradigms or mirror-based VR experiments could be used to target the whole body, allowing exploration of a wider range of symptoms. Comparing third-person to first-person experiences could offer deeper insights into the role of sense of agency. Third, incorporating markers of sense of agency such as sensory attenuation into VR experiments may deepen understanding of its role and the reliability of these markers.^74^

### Attention

#### Attention in FND

Alongside sense of agency, attention is widely theorised to play a fundamental role in FND.^3^ In Bayesian models of perception, attention determines and modulates the relative precision given to both prior expectations and sensory information and thereby in turn the relative weight (precision weighting) of these variables in modulating perceptual states.^53^ For example, in cases of priors with high precision combined with imprecise sensory information, posterior (perceptual) states will be relatively close to the prior beliefs. Therefore, from a Bayesian perspective, attention represents a crucial factor in both the development and persistence of functional symptoms by affording excessive precision to abnormal prior expectations.^53^ This is broadly supported by empirical evidence, for instance, of individuals with FND having a body-focused attentional bias, and consequently displaying increased attention towards symptoms.^3^ Moreover, studies have shown patients with FMD seem to have an abnormal allocation of attention, leading to a cognitive bias in favour of feedforward predictions and a deficit in processing new sensory information.^75^

In a clinical setting, the role of attention is readily demonstrated by worsening of functional symptoms through body-focused attention, whereas distraction generally leads to improvement.^3 76^

#### Attention in VR

Targeting attentional processes is a fundamental aspect of VR and is closely related to the concept of immersion. Immersion entails the subjective feeling of being ‘present’ in a VR environment.^77^ Since real-world visual input is substituted by virtual visual input, a significant degree of attention is diverted to the virtual world, though non-visual sensory input generally remains unchanged and immersion is never all-encompassing. It is however possible to additionally provide auditory, haptic and proprioceptive feedback to create a more immersive experience. Consequently, several VR interventions have been developed to modulate attentional processes, with particular success in pain management.^29^ In addition to distractive interventions, there is some evidence for the ability of VR to alter attentional biases. For example, in social anxiety disorders, where patients have an attentional bias towards external stimuli, a VR intervention resulted in significant reduction of this bias.^78^ Similarly, a reduction in bodily focused attentional bias through VR interventions has been demonstrated in healthy participants.^79^ Furthermore, VR training programmes have been demonstrated to improve attention in patients with ADHD.^80^

#### Attention in VR and FND

Returning to FND, our proposal is that VR can be an effective intervention modality to manipulate attentional processes in patients with FND. For one, these interventions engender distraction. Simple VR distraction methods, which are potentially more comprehensive and immersive than real-life counterparts, could be used to explore the effects of distraction and deepen understanding of the role of attention in symptom expression. A simple experiment would be to monitor severity of FND symptoms during various distractive VR interventions. This could be expanded by incorporating stepped levels of distraction, potentially using multisensory input to explore its effect on symptom severity. This has already been explored in several aforementioned studies such as Gandolfi *et al*’s study of postural stability in FMD during increasingly demanding virtual attentional dual tasks,^43^ as well as several VR studies of PPPD.^46 47 50^ Moreover, adaptive psychophysics could be used to identify the specific level of distraction that engenders symptom attenuation (ie, a symptom attention threshold) and this could be used as an outcome measure in therapy. Second, the use of VR to manipulate attentional biases^78 79^ may elucidate the precise role of attention in FND. By shifting attention towards external stimuli, specifically new sensory inputs provided by the VR system, distorted learning processes and the role of attentional mechanisms could be examined further. In addition, differences between explicit and implicit motor control observed in individuals with FMD could be further explored through the use of VR-based dual tasks to modulate attention.^64^

### Suggestibility

#### Suggestibility in FND

A suggestion can be understood as a communication for a change in awareness or behaviour that is typically experienced as involuntary.^81 82^ Individuals vary considerably in their trait responsiveness to verbal suggestions (direct verbal suggestibility^83^). In a recent meta-analysis, Wieder *et al* 84 found evidence for elevated responsiveness to verbal suggestions in patients with FND compared with healthy controls. The analyses specifically highlighted how patients with FND are especially responsive to symptom-specific provocation methods (suggestive symptom induction), and to a lesser extent, responsive to general suggestions (standardised suggestibility scales). This corroborates the long-held hypothesis that elevated suggestibility plays an important role in FND, with additional implications for the incorporation of suggestion in the treatment of FND.^85 86^ This result also aligns with the Bayesian model of FND, where suggestibility is thought to reflect the tendency to form overly precise priors that are overweighted relative to sensory input.^84 87^ Nevertheless, the specific neurocognitive mechanisms underlying atypical suggestibility in FND remain poorly understood. In particular, it remains unclear whether elevated responsiveness to symptom suggestions exemplifies suggestibility as a risk factor for FND or the result of a kindling or conditioning process whereby repeated symptom experience engenders an attenuation of patients’ symptom thresholds.^59 84^

Within the context of FND, suggestion effects can be reflected in a variety of cues that could be used to provoke, reinforce or reduce symptoms.^85 86^ A previous meta-analysis found that inclusion of suggestion appears to improve treatment outcomes in FND.^88^ However, it should be noted that elevated suggestibility is not unique to patients with FND, nor are all patients with FND suggestible.^84^

#### Suggestibility in VR

Given that a primary feature of VR is to induce visual illusions and changes in experience, there are evident parallels with suggestion effects. In turn, this would imply that elevated suggestibility may translate to increased responsiveness to VR interventions. For instance, individual differences in the RHI are weakly associated with (hypnotic) suggestibility, such that highly suggestible participants experience more sense of ownership over the rubber hand.^89 90^ A recent study^90^ interpreted this association to reflect that the RHI is partly confounded by suggestions, expectations and demand characteristics. Debate continues on the specific role of suggestibility in the RHI and other embodiment experiments, whereby it is accepted that the effects of the RHI are at least partially influenced by suggestions.^91–93^ Hence, it seems reasonable that experimental and clinical manipulations of sense of agency or body ownership should consider the role of suggestibility.

The link between suggestibility and proneness to illusion similarly extends to VR. There is a general association between VR and suggestibility, such that direct verbal suggestibility is associated with the experience of presence in the VR environment.^94^ In addition, demand characteristics have been found to impact on experience of presence and embodiment in VR.^95^ Accordingly, it is plausible that highly suggestible individuals will be more easily immersed in a virtual world.

As for specific interventions, aside from the body ownership illusions, several studies discuss the combination of VR and hypnosis, proposing VR as an augmentation of therapies making use of hypnotic suggestions.^96^ VR hypnosis is an effective analgesic intervention, combining immersion in VR with hypnotic suggestions to modulate pain perception, and seems more effective than VR interventions or hypnosis individually.^97^ However, it remains unclear whether VR shapes therapeutic outcomes through suggestion/placebo effects, induction of dissociative states (eg, depersonalisation) or whether the analgesic effects of VR are simply due to its distractive capacities.^97^

#### Suggestibility in FND and VR

We propose several possibilities as to how VR may serve as a tool for harnessing suggestion in FND. VR can be used to explore the aforementioned relationship between sense of agency and suggestibility, and deepen our understanding of their interaction in FND, using VR-based illusory experiments such as body ownership illusions or VR mirror visual feedback illusions.^37^ Using VR to induce illusory experiences that are not predictable or expected may offer unique opportunities to dissociate demand characteristic effects from other contributing factors. Full body illusions may show promise, since they are impossible in real life and allow for a wide range of suggestive interventions, as opposed to the RHI. Subsequently, comparable experiments may be executed to measure different components of suggestibility in patients with FND, thereby providing more insight into the impact of different types of suggestibility in these patients, for instance, by exploring the role of visual suggestions as opposed to verbal suggestions.^84^ Equally, VR may help to clarify the differences between general and symptom-specific suggestibility.^98^

## Diagnosis

In addition to potentially advancing mechanistic understanding, it is also possible that VR could help develop novel, more reliable ways of diagnosing FND. Existing diagnostic criteria predominantly revolve around the underlying concept of attention, and to a lesser extent, suggestibility.^3^ Manipulating these mechanisms in VR using the aforementioned methods will therefore likely have diagnostic utility.

Due to its potential to create a wide range of environments, scenarios and tasks to facilitate distraction and suggestion, VR could be used to facilitate diagnosis of FND subclasses. For instance, as functional dystonia remains difficult to diagnose, future research could explore the potential of VR to distinguish between functional and non-functional dystonia,^6^ with one possible approach being comparison of susceptibility to VR mirror visual feedback illusions. Another possible experiment with potential diagnostic utility would be that of measuring gait alteration in functional gait disorder when observing an avatar of oneself in VR. In addition, beyond altering audiovisual feedback, changes in haptic feedback through vibrations in VR handheld controllers may merit research on its potential capacity to entrain functional tremors.

Specifically, hypnotic suggestions, as well as visual and verbal suggestions (which can still be effective and administered outside of the context of hypnosis) provided through VR, could be used to provoke functional symptoms, thereby strengthening suggestive symptom induction methods and aiding diagnosis.^99^

In addition, VR could be a promising method for identifying biomarkers for FMD, as previous research has yet to produce a reliable biomarker.^3^ An asset of VR lies in its ability to capture and measure outcome measures, such as movement patterns. Should a VR paradigm capable of influencing functional motor symptoms be developed, specific changes in body movements could be tracked and measured within the virtual environment and used as a quantitative biomarker. Furthermore, VR could aid in further developing other potential biomarkers that have already been identified and hold potential for exploration within VR, such as impaired information processing and abnormal attention.^75 100^

## Treatment

Current treatment options for FND broadly consist of physiotherapy, multidisciplinary rehabilitation and psychotherapy, as well as more novel methods currently under research such as transcranial magnetic stimulation.^7^ Future research should aim to provide additional evidence on current treatment options, deepening understanding of the mechanisms underlying therapeutic effects, and developing new and symptom-specific treatments. We propose several ways in which VR interventions may serve as a promising treatment modality for FND.

### VR as a therapeutic approach for FND

VR could function as an adjunct to existing treatment modalities, such as physiotherapy and rehabilitation.^6^ VR-based rehabilitation has been effectively implemented in other neurological disorders, leading to practical benefits such as cost-effectiveness and accessibility.^13^ VR may in fact expand the domain of rehabilitative interventions, creating training situations that are impracticable in real life. Furthermore, VR may augment existing hypnotic interventions for FND, potentially increasing their efficacy and accessibility.^87 96^ Therefore, VR may contribute to evidence supporting hypnotic interventions in FND, as current evidence remains limited.^86^

The use of VR in combination with brain–computer interface (BCI) technology has demonstrated motor and other rehabilitation potential in stroke and spinal cord injury due to BCI’s capacity for motor imagery-linked functional electrical stimulation of an affected limb (to trigger real movement) as well as the use of motor imagery rehearsal to move an avatar in VR.^101 102^ Similar combined approaches have yet to be studied in FND and may merit exploration.

As outlined previously, VR-based interventions for FND may serve as a low-grade (though alterable) form of exposure given their capacity to provide a relatively stress-free environment, as demonstrated by the extensive study of VR exposure therapy in psychiatric conditions,^20^ thus countering avoidance which is a perpetuating factor in many patients. In addition, the use of VR to create a ‘normal’ movement state rather than that experienced by individuals with FMD could potentially be coupled with the use of certain actions or sensory inputs as a form of conditioning.

**Figure 3:**
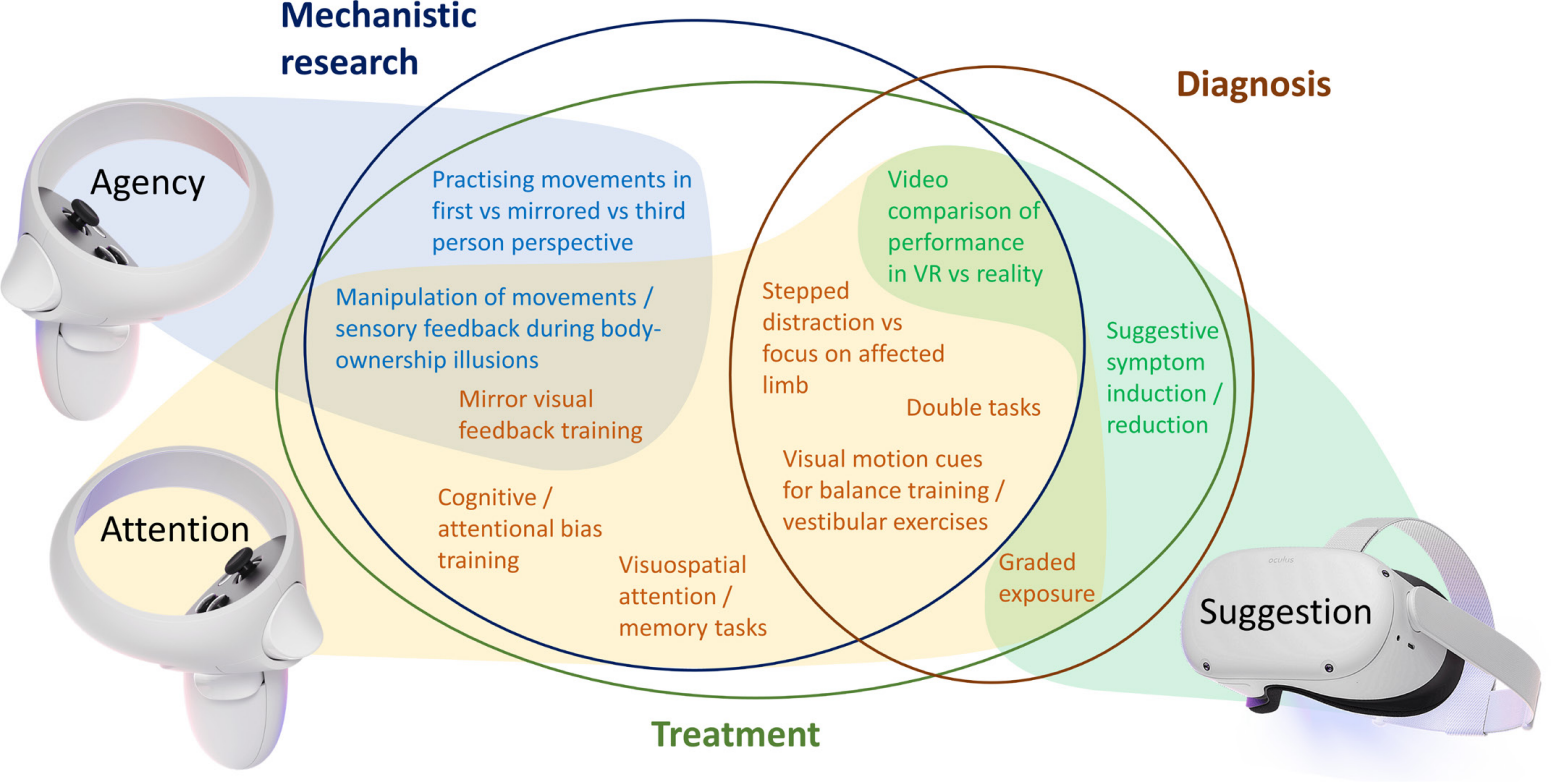
Overview of proposed VR experiments and interventions, their suggested relation to underlying FND mechanisms (shaded) and potential relevance to FND mechanistic research, diagnosis and treatment (circled). Figure created by author HM using images from Oculus Quest 2 press kit under fair use (source: https://about.fb.com/news/2020/09/introducing-oculus-quest-2-the-next-generation-of-all-in-one-vr/). FND, functional neurological disorder; VR, virtual reality.

More importantly, we believe VR may lead to completely new treatment modalities that are not possible in the real world. In our view, these interventions would predominantly rely on simulation, intervening with the predictive coding abnormalities underlying FND, and targeting sense of agency, attention and suggestibility (figure 3). Conducting studies to explore the effect of different body ownership illusions combined with manipulation of sensory feedback (as per Bullock *et al* 41) could pave the path to new treatment modalities. More specifically, VR could aid in developing more symptom-specific interventions, for instance, through tailor-made programmes targeting specific motor symptoms such as tremor or dystonia, or virtual vestibular rehabilitation using visual motion cues and balance training for functional dizziness in PPPD^48 50^ which is already being used in some services to provide individualised virtual interventions based on the patient’s personal triggers,^49^ thereby individualising treatment.^3^

Furthermore, since delivery and acceptance of the FND diagnosis are the (often difficult) first steps of effective treatment, VR may facilitate diagnosis acceptance, contributing to psychoeducation.^103^ Should VR be able to alter FND symptoms through the mechanisms described earlier, video recordings of the patient’s performance could be used to reflect on the objective alteration of symptoms during the VR intervention which (similar to presenting a positive Hoover’s sign) may assist in psychoeducation and acceptance. In addition, a double-blind single-case trial by Ulubas *et al* 104 demonstrated how suggestions may be used to persuade the patient to acknowledge the diagnosis of FND. Given VR’s suggestive potential, similar experiments may be conducted in a VR environment using video recordings to reflect on the experience afterwards and facilitate acceptance of the diagnosis.

Though the majority of VR applications for neurological conditions have focused on rehabilitation-based motor symptom amelioration, approaches taken for VR-based cognitive rehabilitation of working memory in traumatic brain injury may serve as a promising framework for treatment of cognitive symptoms in FND.^105 106^ In addition, functional pain syndromes may potentially respond to VR-based pain distraction, as indicated by altered laser-evoked potential subjective pain rating in patients with migraine depending on the nature of the experienced virtual waiting area.^107^ VR-based behavioural activation may also merit study for FND-related fatigue given its ongoing exploration for use in depression.^108^

More generally, VR-based interventions may improve sustainability and access to treatment due to their potential to be held remotely^109^ or even be delivered through a self-help framework.^108^ This may particularly benefit patients facing difficulties in attending clinics (mobility related or otherwise) though research will be required to determine if either approach would be acceptable or effective in an FND population.

Overall, VR interventions may elucidate the as-of-yet unclear relationship between underlying pathophysiology and observed treatment effects.^6^

## Limitations, pitfalls and concerns for future research

As the ideas we have proposed for future research are largely based on theoretical inferences, it is important to consider potential risks when translating these concepts into practice.

First, it is pertinent to note that not all aspects of FMD treatment can or should be provided through VR, as a broader treatment approach is required when considering psychiatric comorbidity, complex symptoms or extensive disturbance of quality of life. Future studies should not only evaluate VR-based interventions, but also compare them with existing interventions to determine if VR provides a benefit comparable with that conferred in vivo, either as sole treatment or in combination with existing therapeutic modalities.^110^

In addition, it should be noted that some individuals may experience adverse events from VR such as nausea^111^ (although there are methods to minimise this^112^), dissociation^113^ and other symptoms. Further research is required to explore whether augmented/mixed reality-based approaches (in which participants see the real environment around them through a camera but with virtual elements superimposed) could potentially be less disembodying than traditional VR when used for rehabilitation. It is theoretically possible that in some cases, VR-induced dissociative experiences may in fact exacerbate FND symptoms. Therefore, we emphasise that current implementations of VR should be conducted in the presence of a healthcare professional attentive to potential adverse effects, even though VR may eventually create opportunities for home-based treatment.

Third, we should provide a note of caution on the potential effects of altering the pathophysiological mechanisms underlying FMD. Huys *et al* 114 demonstrated that artificially manipulating visual feedback on a computer screen while participants were moving their finger on a mousepad did not improve functional tremor severity, even though one might expect altering sensory feedback would influence functional symptoms. The authors emphasise the potential risk of reinforcing pathological movement patterns when manipulating visual feedback. As we share that concern, we stress that VR interventions should not simply aim to correct visual ‘deficits’, and that manipulating the underlying mechanisms of FMD is complex and demands nuanced, thoughtful interventions.

Fourth, when conducting clinical research using wearable health technology, it is important to consider the ethical implications of data storage and ownership. Such concerns are of particular importance when collaborating with commercial partners to develop VR-based interventions, or when interventions involve movement tracking or collection of other individual data.

Finally, though this theoretical overview has largely focused on the potential applications of VR for motor symptoms and dizziness in FND, this is reflective of the existing literature which is sparse and predominantly composed of such approaches. Given the roles of agency, attention and suggestibility across FND more widely, and existing evidence of VR interventions in cognitive, pain and psychiatric disorders, we believe there is a need for research on VR applications across all FND subtypes, as well as prevalent comorbid symptoms.

## Conclusion

In this review, we have provided a theoretical framework proposing how VR interventions could advance understanding of pathophysiological mechanisms, diagnosis and treatment in patients with FND. Given the lack of empirical evidence on this topic, our proposals are grounded in theoretical inferences, combining the existing literature on FND with research on VR. This review is therefore meant to lay out the groundwork for future clinical research. We believe VR could result in significant advancements in overcoming current challenges in FND research, and subsequently contribute to continuation of the momentum observed in recent decades.^6^
