# Supplementary material for: Virtual reality in functional neurological disorder: a theoretical framework and research agenda for use in the real world
Source: BMJ Neurol Open. 2024 Jul 5;6(2):e000622. doi: 10.1136/bmjno-2023-000622 (PMC11227774; doi:10.1136/bmjno-2023-000622)
Supplement: Supplementary data [file bmjno-2023-000622supp001.pdf]

Supplementary methods

Search Strategy

We first searched the PubMed database for systematic reviews on VR-applications in neurology, psychiatry and specific FND-related disorders such as chronic pain, chronic fatigue syndrome and fibromyalgia. Secondly, to provide a methodological and complete review of the existing literature on FND and VR, we searched the PubMed, MEDLINE, Embase and PsycInfo databases using the keywords ("functional neurological disorder*," or "functional movement disorder*," or "functional motor disorder*," or "conversion disorder*," or "psychogenic*," or "persistent postural-perceptual dizziness*," or "dissociative*," or "psychosomatic*") and ("virtual reality," or "VR," or "augmented reality," or "AR")

The search strategy was conducted in duplicate by authors DB and HM on 15/01/2024. This resulted in 470 studies, 324 after deduplication, 58 after screening titles, 22 after screening abstracts, and 12 relevant studies after screening full texts. Additionally, we searched for clinical trials currently being conducted on Clinicaltrials.gov (condition: functional neurological disorder; other terms: conversion disorder; intervention: virtual reality) and the International Clinical Trials Registry Platform (ICTRP) using similar keywords. This resulted in 2 relevant studies (https://clinicaltrials.gov/study/NCT02764476; https://clinicaltrials.gov/study/NCT05086380).

Inclusion criteria

- Studies with human participants (of any age).
- Participants must have a diagnosis that could be considered a form of functional neurological disorder (unless they are control participants).
- The intervention of interest must be VR and/or AR-based.

Exclusion criteria

- Studies without primary patient data.
